# Intravoxel incoherent motion to differentiate spinal metastasis: A pilot study

**DOI:** 10.3389/fonc.2022.1012440

**Published:** 2022-10-06

**Authors:** Enlong Zhang, Yuan Li, Xiaoying Xing, Siyuan Qin, Huishu Yuan, Ning Lang

**Affiliations:** ^1^ Department of Radiology, Peking University Third Hospital, Beijing, China; ^2^ Department of Radiology, Peking University International Hospital, Beijing, China

**Keywords:** magnetic resonance imaging, metastasis, spine, diffusion, intravoxel incoherent motion

## Abstract

**Background:**

To investigate the value of intravoxel incoherent motion (IVIM) magnetic resonance imaging (MRI) to discriminate spinal metastasis from tuberculous spondylitis.

**Methods:**

This study included 50 patients with spinal metastasis (32 lung cancer, 7 breast cancer, 11 renal cancer), and 20 with tuberculous spondylitis. The IVIM parameters, including the single-index model (apparent diffusion coefficient (ADC)-stand), double exponential model (ADC_slow_, ADC_fast_, and f), and the stretched-exponential model parameters (distributed diffusion coefficient (DDC) and α), were acquired. Receiver operating characteristic (ROC) and the area under the ROC curve (AUC) analysis was used to evaluate the diagnostic performance. Each parameter was substituted into a logistic regression model to determine the meaningful parameters, and the combined diagnostic performance was evaluated.

**Results:**

The ADC_fast_ and f showed significant differences between spinal metastasis and tuberculous spondylitis (all p < 0.05). The logistic regression model results showed that ADC_fast_ and f were independent factors affecting the outcome (P < 0.05). The AUC values of ADC_fast_ and f were 0.823 (95% confidence interval (CI): 0.719 to 0.927) and 0.876 (95%CI: 0.782 to 0.969), respectively. ADC_fast_ combined with f showed the highest AUC value of 0.925 (95% CI: 0.858 to 0.992).

**Conclusions:**

IVIM MR imaging might be helpful to differentiate spinal metastasis from tuberculous spondylitis, and provide guidance for clinical treatment.

## Introduction

Osteoporosis, trauma, infection, or tumors are typical causes of clinically common diseases of the spine. Their diagnosis mainly depends on imaging findings and clinical features. However, benign and malignant lesions in the spine might have similar imaging findings, especially in the early stages of the disease (1). In addition, with the increasing incidence of cancer in modern society, the diagnosis of spinal bone lesions has become more difficult. The spine is a site of metastasis in 10–15% of cancers, making it the third most common site for cancer cells to metastasize (2) and spinal metastases are the most common tumors of the spine (3). Spinal metastases are commonly caused by lung cancer, breast cancer, and renal cancer. The major differential diagnosis for spinal metastases is spinal tuberculosis. Spinal metastases and tuberculosis with typical and specific imaging findings are relatively easy to diagnose correctly. However, the clinical presentations between them are usually not characteristic, leading to misdiagnosis. Besides, the imaging features of different diseases have some overlap. Therefore, an effective differential diagnosis method to identify different lesions in the spine is of great significance in clinical practice (4).

Intravoxel incoherent motion (IVIM) is a noninvasive magnetic resonance imaging (MRI) method to visualize microscopic motions of water, which refers to translational motion that presents a velocity direction and/or amplitude distribution within a given voxel and over a measured time (5). Water molecular diffusion and perfusion-related diffusion can be achieved by allowing monitoring of the extraction of molecular diffusion coefficients (ADC_slow_), perfusion-related diffusion (ADC_fast_), and the perfusion fraction (f). Recently, IVIM has been used to estimate tissue water diffusivity and micro-capillary perfusion. Perfusion MRI based on IVIM, which does not require contrast agents, has been widely used in studies of oncology. IVIM has been used for glioma grading, tumor diagnosis, central nervous system and abdomen since its development (6–10).

It was reported that IVIM based on the biexponential model might help to differentiate malignant spinal tumors from acute vertebral compression fractures and tuberculous spondylitis (4). However, only seven patients with tuberculous spondylitis and eight patients with malignant spinal tumors were included in the study, including lymphoma, myeloma, and metastatic tumors (primary tumors included lung cancer, prostate cancer and large cell neuroendocrine cancer). The results showed that the ADC_slow_ and ADC_fast_ values of malignant spinal tumors were significantly different from those of tuberculous spondylitis (all P < 0.05) (4). In addition, IVIM has certain clinical value in differentiating spinal metastasis from myeloma and atypical vertebral hemangiomas (11, 12). IVIM-diffusion-weighted imaging (DWI) could allow the early diagnosis of ductal carcinoma *in situ* of the breast, and reduce the misdiagnosis and over-treatment of benign lesions (13). IVIM and conventional radiological features improve the preoperative assessment of microvascular invasion in patients with hepatocellular carcinoma (14). IVIM-DWI-derived parameters, especially the pseudo diffusion coefficient, were related to tumor grade and stage in patients with rectal cancer, and the difference between subjects with extramural vascular invasion and those without extramural vascular invasion was statistically significant. IVIM-DWI derived parameters could also help to predict tumor aggressiveness and prognosis (15). In addition, IVIM-DWI can predict the overall survival rate of newly diagnosed acute myeloid leukemia (16). IVIM MRI quantitatively measures local microvascular muscle perfusion to detect muscle activation patterns through walking and running (17). Zhu et al. reported that IVIM with certain b values (0, 50, 200, 1000) collects diffusion and perfusion information from a single short MRI sequence, which might have important implications for the imaging of patients with acute ischemic stroke (18). Accordingly, the aim of the present study was to investigate the IVIM parameters in spinal diseases with the hope of discriminating spinal metastasis from tuberculous spondylitis.

## Methods

### Patient selection

From January 2016 to October 2018, 70 consecutive patients were enrolled in this study, and IVIM MRI was performed at 3.0T before treatment and puncture. Among them, 50 patients had spinal metastasis (32 with lung cancer, 7 with breast cancer, and 11 with kidney cancer) and 20 patients had tuberculous spondylitis. The inclusion criteria were: Clinically definitive diagnosis or histopathological diagnosis. The exclusion criteria were as follows: 1) A history of interventional therapy such as radiotherapy, chemotherapy, or radiofrequency ablation before examination; 2) serious artifacts in the image; 3) the presence of smaller lesions (< 1 cm in diameter), resulting in inaccurate measurements; and 4) no clinical diagnosis.

### MRI acquisitions

Scanning was performed using a 3.0T MRI system (GE Healthcare 750, Chicago, IL, USA) and 8-channel spinal coils. Conventional MRI sequence scanning was performed to help lesion localization. For patients with multiple lesions, lesions with the largest diameter were selected for DWI scanning. Conventional MRI sequences included axial and sagittal fast recovery fast spin echo (FRFSE) sequence (T2 WI) with a repetition time (TR) of 2800 ~ 4341 ms, echo time (TE) of 98–142ms, a slice thickness of 3.0 mm, a slice gap of 0.3mm, a band width of 62.5 kHz, a field of view (FOV) of 20 cm × 20 cm to 36 cm × 36 cm, and a matrix of 288 × 288 (axial) and 488 × 320 (sagittal). The sagittal FSE sequence T1WI FSE had a TR of 500–642 ms, a TE of 8–11 ms, a slice thickness of 3. 0 mm, a slice gap of 0. 3 mm, a bandwidth of 62.5 kHz, an FOV of 28 cm × 28 cm to 36 cm × 36 cm, and a matrix of 320 × 320. The cervical and thoracic sagittal position IDEAL sequence T2 WI had a TR of 3000 ms, a TE of 69 ms, a slice thickness of 30 m, a slice gap of 0.3 mm, a band width of 833 kHz, an FOV of 28 cm × 28 cm to 36 cm × 36 cm; and a matrix of 320 × 192. The lumbar and sacro coccyx lipid suppression FRFSE sequence T2 WI had a TR of 2409–3100 ms, a TE of 88–98 ms, a slice thickness of 3. 0 mm, a slice gap of 0. 3 mm, a bandwidth of 50.0 kHz, and an FOV of 28 cm × 28 cm to 36 cm × 36 cm. IVIM-DWI was obtained with an axis single excitation spin-echo echo-planar imaging sequence, with the following parameters: a TR of 3000 ms, a TE of 64 ms, a slice thickness of 4.0 mm, a layer spacing of 0.4 mm, a bandwidth of 250. 0 kHz, an FOV of 24 cm × 24 cm, and a matrix of 128 × 64. The b values were 0, 20, 50, 100, 150, 200, 400, 800, 1200, and1500 s/mm^2^. The scanning time was 252 s.

### Regions of interest

The region of interest (ROI) was set in the lesion center where the cross-section showed the largest level of the tumor with an area of 30–100 mm^2^ (11, 19) and the solid components of the tumor were included avoiding the lesion edge, necrosis, bone cortex of vertebral bodies or attachments, and blood vessels. The analysis was performed independently by two radiologists (initials: ELZ, XYX) with more than 5 years of experience on the GE AW4.5 workstation, and the average of the ROI measurements for each parameter was taken as the final measurement result.

### Post-processing and MR Imaging analysis

**Figure 1:**
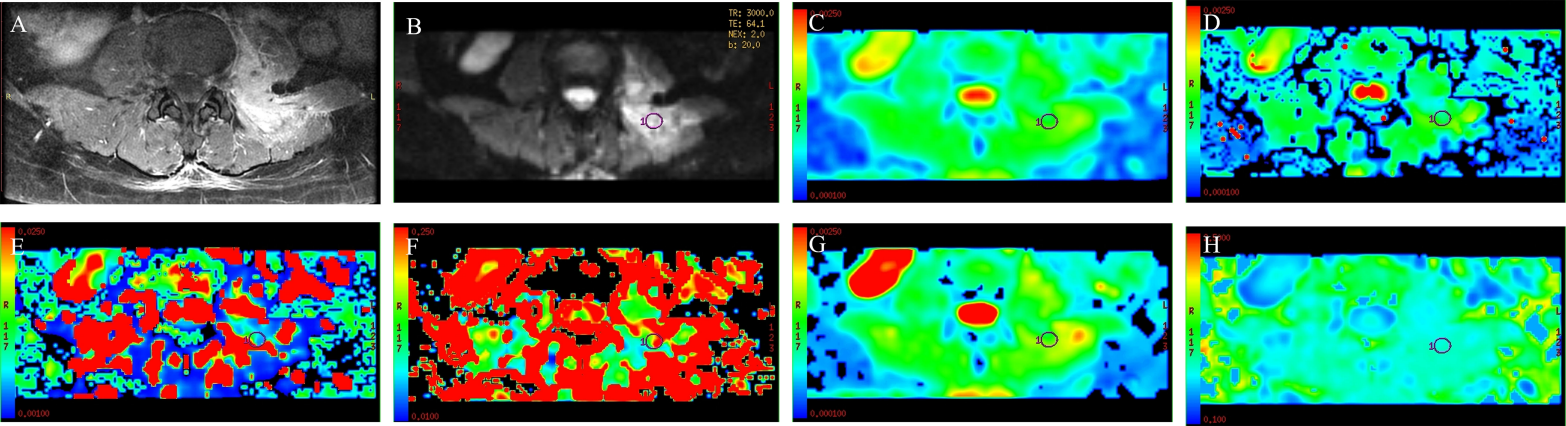
A 53-year-old man with spinal metastasis from lung cancer. The lesion showed obvious enhancement on T1WI **(A)** and hyperintensity on DWI **(B)**. ADC values were 0.926 × 10^−3^ mm^2^/s **(C)**. The ROI was placed on DWI **(B)** and copied to the other IVIM parametric maps of ADC_slow_, ADC_fast_, f, DDC, and α **(D–H)** with values of 0.8 × 10^−3^ mm^2^/s, 0.0211 mm^2^/s, 0.139, 0.945 × 10^−3^ mm^2^/s, and 0.759, respectively.

**Figure 2:**
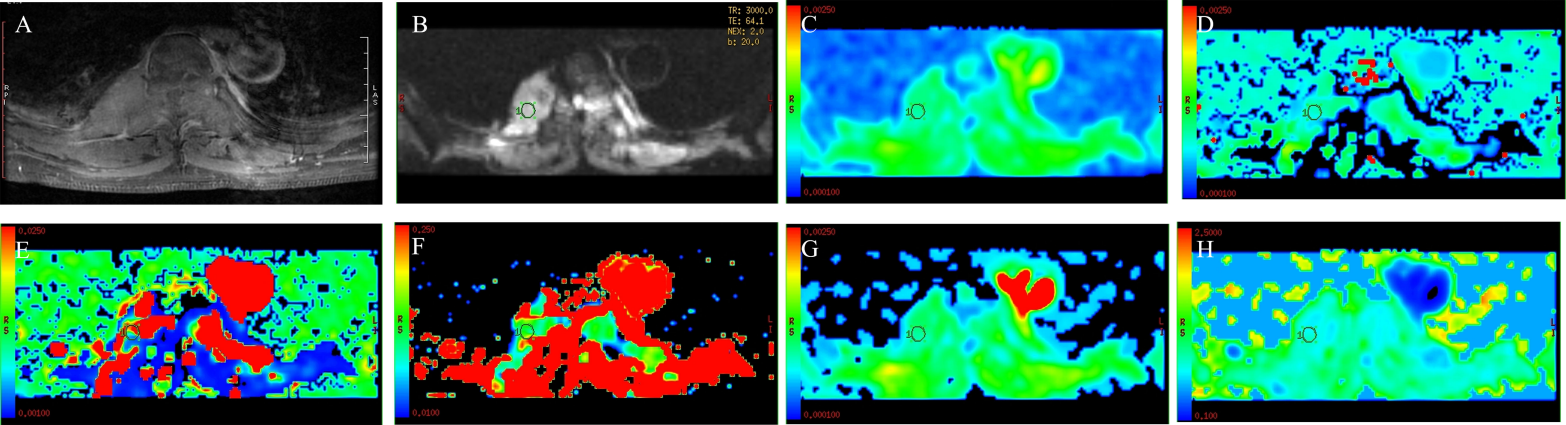
A 44-year-old woman with spinal tuberculosis. The lesion showed obvious enhancement on T1WI **(A)** and hyperintensity on DWI **(B)**. ADC values were 1.28 × 10^−3^ mm^2^/s **(C)**. The ROI was placed on DWI **(B)** and copied to the other IVIM parametric maps of ADC_slow_, ADC_fast_, f, DDC, and α **(D–H)** with values of 1.11 × 10^−3^ mm^2^/s, 0.0131 mm^2^/s, 0.190, 1.41 × 10^−3^ mm^2^/s, and 0.807, respectively.

All data were transferred to an imaging workstation for analysis. The IVIM parameters, including single-index model (Apparent diffusion coefficient (ADC)-stand), double exponential model (ADC_slow_, ADC_fast_, and the perfusion fraction (f)) and stretched-exponential model parameters (distributed diffusion coefficient (DDC) and intravoxel water diffusion heterogeneity (α)) were acquired. Two radiologists measured these parameters separately for each lesion by drawing an ROI (**Figures 1**, **2**). Receiver operating characteristic (ROC) and the area under the ROC curve (AUC) analysis was used to evaluate the diagnostic performance.

The ADC value was obtained by using high and low b values that were monoexponentially fitted to the following equation: Sb = S0 × exp (-b × ADC), where Sb is the signal intensity observed in the absence of a diffusion gradient. The formula for the biexponential model was as follows: Sb = S0 × [(1- f) × exp (-b × ADC_slow_) + exp (b× ADC_fast_)], where S0 is the mean signal intensity, ADC_slow_ is the molecular diffusion coefficient, ADC_fast_ is the perfusion-related coefficient, and f is the perfusion fraction. The formula for the stretched-exponential model was as follows: Sb = S0 × exp (-b × DDCα), where α reflects the diffusion heterogeneity of water molecules in voxels.

### Statistical analysis

All statistical analyses were performed using SPSS version 22.0 (IBM Corp., Armonk, NY, USA). Continuous variables are expressed as the mean ± standard deviation (SD). The intraclass correlation coefficient (ICC) was used to analyze the agreement between the two radiologists and was interpreted as follows: 0.00–0.20, poor agreement, 0.21–0.4, fair agreement, 0.41–0.60, moderate agreement, 0.61–0.80, good agreement, and 0.81–1.00, excellent agreement. Categorical variables are expressed as a percentage with a 95% confidence interval (CI). Two independent sample t-tests were used to compare normally distributed data, and the Mann-Whitney U test was used to compare non-normally distributed data. The variables with statistical significance in univariate analysis were included in the multivariate logistic regression model. Nonparametric ROC analysis was performed to evaluate the diagnostic performance of the IVIM parameters. The AUC was calculated to investigate the performance of these parameters, and the cut-off values with the largest sum of sensitivity and specificity were calculated from the ROC curves. The significance level set at p < 0.05.

## Results

### Patients

There were 50 patients with spinal metastasis (32 with lung cancer, 7 with breast cancer, 11 with renal cancer), with a mean age ± SD of 56.92 ± 9.29 years; and 20 patients with tuberculous spondylitis (means age ± SD: 48.82 ± 19.84 years). The participants’ basic information is provided in **Table 1**. **Figures 1** and **2** show IVIM imaging and the measurement methods for representative cases of spinal metastasis from lung cancer and tuberculous spondylitis.

**Table 1:** Patient demographics and IVIM parameters among patients with tuberculous spondylitis and spinal metastasis.

Patient group	No. of patients	Male	Female	Mean age (y)^a^	ADC_stand_(×10^−3^ mm^2^/s)	ADC_slow_(×10^−3^ mm^2^/s)	ADC_fast_(×10^−3^ mm^2^/s)	f	DDC(×10^−3^ mm^2^/s)	α
Tuberculous spondylitis	20	11	9	48.82 ± 19.84	1.11 ± 0.25	0.77 ± 0.35	21.96 ± .782	0.45 ± 0.20	1.22 ± 0.34	0.77 ± 0.10
Spinal metastasis	50	30	20	56.92 ± 9.29	1.05 ± 0.27	0.85 ± 0.28	33.87 ± 6.87	0.25 ± 0.15	1.17 ± 0.44	0.75 ± 0.09
lung cancer	32	20	12	57.66 ± 9.40	0.95 ± 0.23	0.73 ± 0.23	33.31 ± 5.44	0.27 ± 0.18	0.99 ± 0.29	0.78 ± 0.07
breast cancer	7	0	7	53.29 ± 9.55	1.20 ± 0.30	1.05 ± 0.29	36.96 ± 10.45	0.22 ± 0.14	1.60 ± 0.50	0.75 ± 0.08
renal cancer	11	10	1	57.09 ± 9.12	1.25 ± 0.23	0.82 ± 0.30	30.46 ± 8.93	0.31 ± 0.19	1.19 ± 0.41	0.76 ± 0.09

a Data are means ± standard deviations.

### Interobserver reproducibility for MRI measurement

Excellent interobserver (initials: EZ and XX) reproducibility was obtained for ADC_stand_, ADC_slow_, ADC_fast_, f values, DDC and α, with ICC values of 0.929 (95% CI: 0.889 to 0.956), 0.946 (95% CI: 0.914–0.966), 0.849 (95% CI: 0.768 to 0.903), 0.868 (95% CI: 0.796 to 0.916), 0.924 (95% CI: 0.880 to 0.952), and 0.911 (95% CI: 0.860 to 0.944), respectively.

### Comparison of IVIM parameters among different groups

The mean values of ADC_stand_, ADC_slow_, ADC_fast_, f, DDC, and α of tuberculous spondylitis and spinal metastasis are shown in **Table 1**. The results showed the ADC_fast_ of spinal metastasis was significantly higher compared with that of tuberculous spondylitis, and the f values in patients with spinal metastasis were lower compared than those in patients with tuberculous spondylitis (all p < 0.05) (**Table 2**). The mean ADC_stand_, ADC_slow_, DDC, and α values between spinal metastasis and tuberculous spondylitis did not reach statistical significance.

**Table 2:** Comparison of mean IVIM-parameters for tuberculous spondylitis and spinal metastasis.

Parameters	t	*p*
ADC_stand_	0.855	0.395
ADC_slow_	-1.017	0.313
ADC_fast_	-6.296	< 0.0001**
f	4.645	< 0.0001**
DDC	0.399	0.691
α	0.854	0.396

**p < 0.0001.

### Logistic regression model

The variables with statistical significance in the univariate analysis and those that were considered to have influence on the outcome were included in the multivariate logistic regression model. The results showed that ADC_fast_ and f were independent variables affecting the outcome (p < 0.05) (**Table 3**).

**Table 3:** The results of logistic regression analysis.

Parameters	B	S.E.	Wald	*p*	OR	95% CI OR	
ADC_fast_	−6.185	2.408	6.597	0.010*	0.002	0.001	0.231
f	0.235	0.068	11.847	0.001*	1.265	1.107	1.446

*p < 0.05.

### ROC-analysis

**Figure 3:**
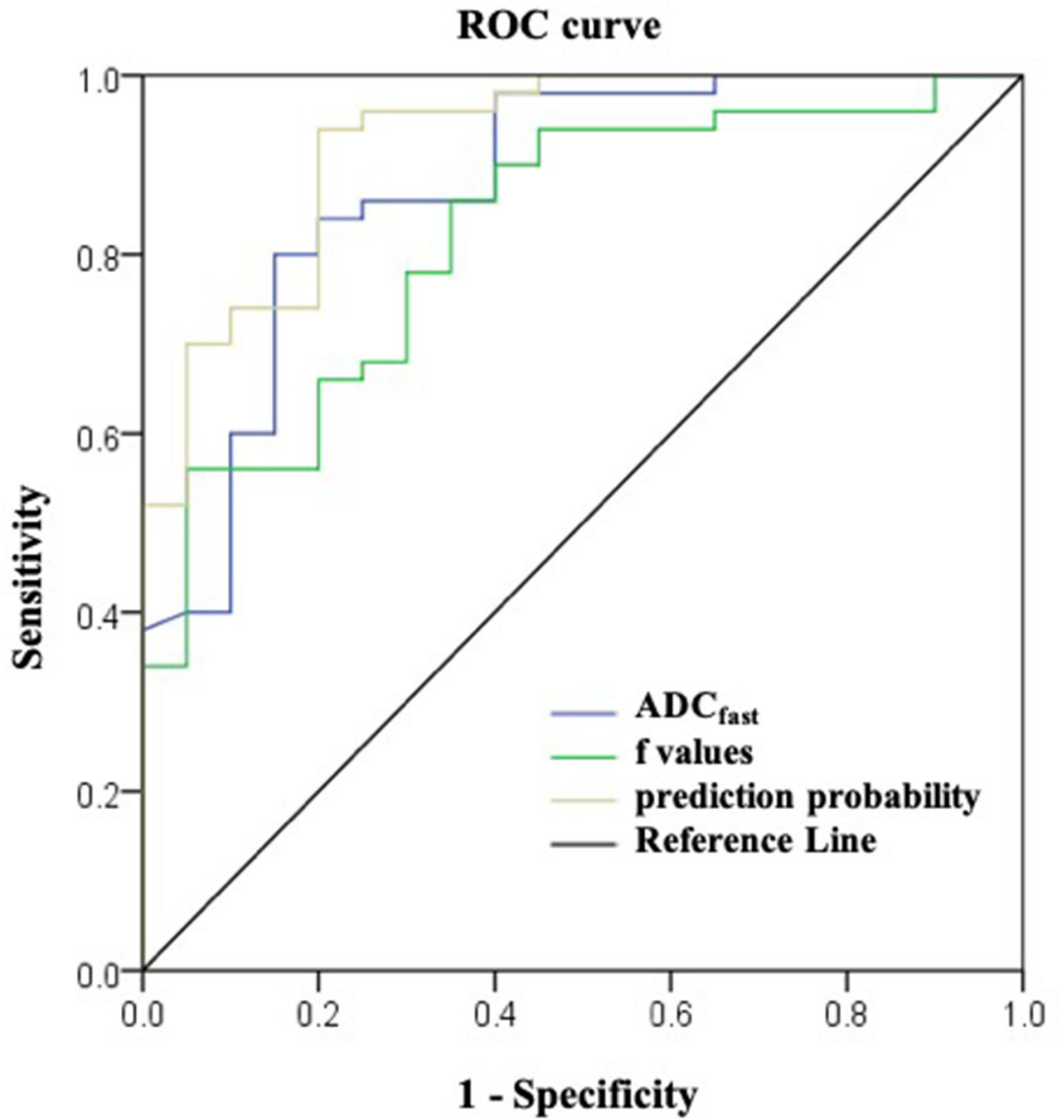
ROC curves to differentiate tuberculous spondylitis and spinal metastasis.

In the ROC-analysis for the differentiation of spinal metastasis from tuberculous spondylitis, the AUC values of ADC_fast_ and f were 0.876 (95% CI, 0.782 to 0.9969) and 0.823 (95% CI, 0.719 to 0.927), respectively. The sensitivity and specificity of the ADC_fast_ value to differentiate spinal metastasis from tuberculous spondylitis were 80.0% and 85.0%, respectively. The sensitivity and specificity of the f value were 86.0% and 65.0%. ADC_fast_ combined with f showed much higher AUC than ADC_fast_ and f, which the AUC values were 0.925 (95% CI, 0.858 to 0.992). Using the ADC_fast_ combined with f as an index to discriminate spinal metastasis from tuberculous spondylitis, the sensitivity and specificity were 94.0% and 80.0%, respectively (**Table 4**, **Figure 3**).

**Table 4:** Efficacy of IVIM in the differential diagnosis of tuberculous spondylitis and spinal metastasis.

Parameters	AUC (95% CI)	Cut-off values	Sensitivity (95% CI)	Specificity (95% CI)
ADC_fast_	0.876 (0.782–0.969)	> 29.05	80.0 (66.3–90.0)	85.0 (62.1–96.6)
f	0.823 (0.719–0.927)	< 0.4	86.0 (73.3–94.2)	65.0 (40.8–84.5)
ADC_fast_ and f	0.925 (0.858–0.992)		94.0 (83.4–98.7)	80.0 (56.3–94.1)

## Discussion

The IVIM method is a diffusion-weighted MRI sequence used to estimate perfusion parameters, which has several advantages over commonly used methods. It is a non-invasive alternative to perfusion measurement, eliminating the need for intravenous injection of exogenous contrast agent through a single image sequence, thereby reducing the examination time. In addition, its signal is highly spatially specific because it comes primarily from a place where measurements are taken independently of the arterial blood flow path. Finally, it provides additional information compared to Arterial spin labelling (ASL), and the combination of the two approaches can be used to assess neurological diseases (20).

Recently, it was reported that the IVIM parameters could help to assess the early diagnosis and differentiation of diseases more precisely, as well as quantitatively monitor the effectiveness of treatment for tumors and other diseases. Ding et al. (21) compared the diagnostic values of IVIM, conventional DWI, and diffusion kurtosis imaging (DKI) to differentiate between benign and malignant renal tumors. They found that the D value was the best parameter to differentiate cell renal cell carcinoma (ccRCC) from benign renal tumors. The f value was the best parameter to differentiate non-ccRCC from benign renal tumors. IVIM parameters had the best performance, while the DWI and DKI parameters had similar performances in differentiating malignant and benign renal tumors. Another study found that rectal cancers with different KRAS mutation statuses had distinctive diffusion/perfusion characteristics. The D values were lower in the KRAS mutant group. A higher D* value was demonstrated in the KRAS mutant group. IVIM MRI might potentially help to predict preoperative KRAS mutant status (22). Zhu et al. (23) evaluated the performance of tumor size and IVIM-derived parameters to predict long-term prognosis, and found that IVIM MR imaging has great potential to predict long-term prognosis in patients with advanced cervical cancer treated with concurrent chemo-radiotherapy.

In early or atypical spinal tuberculosis, there is no typical obvious bone erosion or abscess, and the imaging findings are complex and are sometimes similar to tumors, leading to misdiagnosis. While this is not common, it is still necessary to find ways to avoid it. Within the spinal column, metastasis is more commonly found in the thoracic region, followed by the lumbar region, while the cervical region is the least likely place to find metastasis. The aim of this study was to evaluate the diagnostic performance of IVIM MRI to differentiate spinal metastasis. To the best of our knowledge, our investigation is the first to illustrate the use of IVIM parameters to differentiate spinal tuberculosis from spinal metastasis.

Our results showed that some IVIM parameters could help to discriminate spinal metastasis from tuberculous spondylitis and could investigate the feasibility of tumor type differential diagnosis of different metastases, including lung cancer, breast cancer, and renal cancer. Conventional DWI is based on the micro-movement of water molecules, which reflects the speed of water diffusion in the tissue. ADC values can quantitatively evaluate tissue diffusion and show microscopic changes at the cellular level caused by pathophysiological changes. In our study, no significant difference in ADC values was found between spinal metastases and tuberculous spondylitis, suggesting an ADC overlap in distinguishing them. Therefore, the results showed that the role of traditional ADC DWI in differentiating benign and malignant lesions of the spine is limited, which is consistent with previous studies (4, 24, 25).

The ADC_slow_ is mainly affected by water molecule diffusion of the lesion tissue and ADC_fast_ is mainly affected by capillary microcirculation perfusion. Our results showed the ADC_fast_ value of spinal metastases was significantly higher than that of tuberculous spondylitis, which suggested that perfusion is greatly increased. However, no significant differences were found between the ADC_slow_ values between them. However, the ADC_slow_ value of spinal metastasis with lung cancer was significantly lower than that of spinal metastasis with breast cancer as well as with renal cancer, suggesting that true diffusion is more restricted in lung cancer than in breast cancer and renal cancer. ADC_fast_ and f values, but not the ADC_slow_ value, showed better diagnostic performance than ADC values to differentiate spinal metastasis from tuberculous spondylitis. The f value is mainly affected by the blood volume of microcirculation perfusion, reflecting the proportion of microcirculation perfusion in tissue diffusion. Our results showed that the f value of spinal metastasis was lower than that of tuberculous spondylitis. Similar findings were reported in nasopharyngeal, pancreatic, and cervical lymph node metastases (26, 27). The reasons might be the high cell density of spinal metastasis, compression of the microvessels in the intercellular stroma, and the compression, deformity, and disordered branching of new vessels in malignant lesions, leading to a decrease in the proportion of microperfusion components. In our study, ADC_fast_ combined with f showed a much higher AUC than ADC_fast_ or f alone. These finding suggested that ADC_fast_ combined with f was more valuable for the differential diagnosis of spinal metastasis and tuberculous spondylitis.

The DDC and α value reflect the heterogeneity of diffusion in the tissue. The value of α is set between 0 and 1. The closer the α value is to 1, the higher the homogeneity of the diffusion component; the closer the α value is to 0, the higher the heterogeneity of the diffusion component and the more complex the diffusion component. In this study, the DDC and α values of spinal metastasis was lower than that of tuberculous spondylitis; however, the difference was not significant. This indicated that compared with tuberculous spondylitis, malignant tissue is more complex and heterogeneous, which leads to decreases in the DDC and α values.

Although our findings were novel, our study also has some limitations, which we will improve in future work. Firstly, the sample size of patients with spinal metastasis and tuberculous spondylitis was small, which might have lowered our confidence in the statistical results. Secondly, all values were measured by manual outlining of the ROI, and the ROI was placed on the solid components of the tumor to calculate the average value, which might have introduced errors. Although it was representative to some extent, it was not conducive to the evaluation of tumor heterogeneity. Thirdly, IVIM technology itself is not stable. So far there is no specific standard regarding multiple b values of IVIM sequences, and the calculations still need further exploration (28).

In conclusion, IVIM MR imaging might be helpful to differentiate spinal metastasis from tuberculous spondylitis and provide guidance for clinical treatment. The combination of ADC_fast_ and f parameters was better than ADC_fast_ or f alone. Despite the small patient population, this study might lead to further developments in the application of IVIM to differentiate benign from malignant spinal skeletal lesions. In addition, emerging technologies, such as radiomics and deep learning algorithms, might contribute to the diagnosis of spinal lesions.

## Data availability statement

The original contributions presented in the study are included in the article/supplementary material. Further inquiries can be directed to the corresponding authors.

## Ethics statement

The studies involving human participants were reviewed and approved by Peking University Third Hospital Medical Science Research Ethic Committee. The patients/participants provided their written informed consent to participate in this study. Written informed consent was obtained from the individual(s) for the publication of any potentially identifiable images or data included in this article.

## Author contributions

All authors contributed extensively to the work presented in this paper. Their roles are given below. Design of the work: NL. Acquisition of data: YL, XX. Interpretation of data: EZ, SQ. Drafting/Critical revision: EZ, YL, HY. Final approval: NL.

## Funding

This study has received funding by National Natural Science Foundation of China (No. 81971578; 81901791); Beijing Natural Science Foundation (No. Z190020); Peking University International Hospital Research Grant (No. YN2019QN03).

## Conflict of interest

The authors declare that the research was conducted in the absence of any commercial or financial relationships that could be construed as a potential conflict of interest.

## Publisher’s note

All claims expressed in this article are solely those of the authors and do not necessarily represent those of their affiliated organizations, or those of the publisher, the editors and the reviewers. Any product that may be evaluated in this article, or claim that may be made by its manufacturer, is not guaranteed or endorsed by the publisher.

## Abbreviations

IVIM, Intravoxel incoherent motion; ADC, Apparent diffusion coefficient; DDC, Distributed diffusion coefficient; ROC, Receiver operating characteristic; AUC, Areas under the curves; ICC, Intraclass correlation coefficient; CI, Confidence interval; f, Perfusion fraction; TR, Repetition time; TE, Echo time; FOV, Field of view; ROI, Region of interest; DWI, Diffusion-weighted imaging; DKI, Diffusion kurtosis imaging; ccRCC, Cell renal cell carcinoma.
